# PCE3 Plays a Role in the Reproduction of Male *Nilaparvata lugens*

**DOI:** 10.3390/insects12020114

**Published:** 2021-01-28

**Authors:** Rong-er Zheng, Jinliang Ji, Jiamin Wu, Ruijuan Zhang, Yabin Li, Xiaoping Yu, Yipeng Xu

**Affiliations:** Zhejiang Provincial Key Laboratory of Biometrology and Inspection & Quarantine, China Jiliang University, Hangzhou 310018, China; qq651047935@163.com (R.-e.Z.); j1793505637@163.com (J.J.); wujiamin94@163.com (J.W.); 18893781226@163.com (R.Z.); lyb13372418590tt@163.com (Y.L.); yxp@cjlu.edu.cn (X.Y.)

**Keywords:** *Nilaparvata lugens*, proclotting enzyme, RNAi, reproduction

## Abstract

**Simple Summary:**

The brown planthopper (BPH), *Nilaparvata lugens*, is one of the most harmful rice crop pest insects. The use of RNAi is a feasible strategy for controlling this pest. In this study, we evaluated the importance of *PCE3* in the development and reproduction of male BPH. We found that *PCE3* could regulate the development of the male internal genitalia and reduce the oviposition level of the females that mated with males treated with dsRNA targeting the *N. lugens PCE3* gene, causing eggs not to hatch. Our findings indicate that *PCE3* is an important gene in regulating male fecundity and a promising target for controlling BPH.

**Abstract:**

*Nilaparvata lugens* proclotting enzymes (*Nl*PCEs) belong to the clip domain serine protease (clip-SP) family, which is a characteristic protease family in arthropods. *NlPCE3* was previously reported to regulate egg production and development in female *N. lugens*, but its role in male *N. lugens* is unclear. In the present study, qPCR analysis showed that *NlPCE3* was expressed in three different tissues (gut, testis and fat body). RNAi revealed that ds*NlPCE3* injection made the male vas deferens thinner and reduced the oviposition level of the females that mated with ds*NlPCE3*-treated males, causing eggs not to hatch. Furthermore, immunofluorescence staining showed that *Nl*PCE3 was widely expressed in the male internal genitalia. However, after ds*NlPCE3* injection, expression of *Nl*PCE3 was diffuse in the male internal genitalia, whose peripheral cells seemed degraded. Overall, these results indicate that *NlPCE3* is important for reproduction in male *N. lugens*.

## 1. Introduction

The serine protease (SP) and serine protease homolog (SPH) family is one of the most characteristic enzyme families, playing vital roles in the process of digestion, blood clotting and immunity [1,2]. Certain SPs and SPHs containing one or more disulfide-bridged structures named clip domains are called clip serine proteases (clip-SPs) [3]. In contrast to other SPs, clip-SPs are non-digestive. In addition to one or more N-terminal clip domains, clip-SPs contain a C-terminal S1A family SP domain and a linker sequence [3]. The clip domain may regulate protein–protein interaction similarly to other motifs that are found in SPs, such as low-density lipoprotein receptor class A repeat, scavenger receptor cysteine-rich domain and thrombospondin 2 domain. The clip domain may also anchor enzymes to the surface of invaded organisms by participating in a cascade pathway involving SP-related proteins. The C-terminal S1A family SP domain usually adopts a chymotrypsin-like fold, which consists of two adjacent β-barrel-like structures arranged perpendicularly to each other [4]. This C-terminal domain contains a His-Asp-Ser catalytic triad that is responsible for the acyl transfer [5]. Clip-SPs are divided by phylogenetic analysis into four groups named clip-A, clip-B, clip-C and clip-D [6]. According to sequence similarity and the distances between six highly conserved cysteines, clip-SPs can be further divided into group 1a, group 1b, group 2 and group 3 [7], and they are related to clip-C, clip-D, clip-B and clip-A, respectively [8]. 

To date, clip-SPs have been found only in arthropods such as *Acyrthosiphon pisum*, *Drosophila melanogaster*, *Manduca sexta*, *Scylla paramamosain*, *Eriocheir sinensis* and *Apis mellifera*. Clip-SPs in arthropods have been reported functioning in the control of dorsoventral patterning during fertilization or embryonic development [1,9,10,11] and participating in various aspects of innate immune regulation, including the regulation of specific activation of prophenoloxidase required for melanization response [12,13,14], hemolymph blood clotting function [15], defense responses to microbial infection mediated by activation of the Toll pathway [1] and antibacterial activity [16,17,18].

The brown planthopper (BPH), *Nilaparvata lugens* (Hemiptera: Delphacidae), is one of the most destructive rice plant pests in China and Southeast Asia. BPH is an *r*-selected species with high fertility and is difficult to effectively control. *PCE3* is one clip-SP gene found in *N. lugens* (*NlPCE3*). In a previous study, we demonstrated that *NlPCE3* could be a target for controlling BPH as it has an important role in female reproduction, including reduced egg production, hatching rate and ovarian development [19]. In addition, the experimental data showed that *NlPCE3* has a substantially high expression level in male adults, suggesting that it may also play important roles in males. In the present study, we further examine the function of *NlPCE3* in male BPH to better elucidate the functions of this enzyme.

## 2. Materials and Methods 

### 2.1. Culture of Insects

*N. lugens* used in this study was maintained in a climatron at China Jiliang University, Hangzhou, China. The insects were reared on rice seedlings (Taichung Native 1) at 27 ± 1 °C with 60% humidity under a 16 h light: 8 h dark photoperiod. 

### 2.2. Real-Time Quantitative PCR Analysis

The expression level of *NlPCE3* in male BPH samples was analyzed using real-time quantitative PCR (qPCR). Total RNA from males was extracted with a TaKaRa MiniBEST Universal RNA Extraction Kit (TaKaRa, Dalian, China), and the cDNA template for qPCR was synthesized with a Perfect Real Time PrimeScript™ RT reagent Kit with a gDNA Eraser (TaKaRa). The quality of extracted RNA was determined using a NanoDrop 2000 spectrophotometer (Thermo Fisher Scientific, Waltham, MA, USA) and confirmed with agarose gel electrophoresis. The qPCR reagent was SYBR^®^ Premix ExTaq™ II (Tli RNaseH Plus) (TaKaRa). The special primers for *NlPCE3* qPCR were q*NlPCE3-*F and q*NlPCE3*-R, and 18S rRNA was used as the qPCR internal reference, with primers q*Nl*18s-F and q*Nl*18s-R (Supplementary Table S1) [19]. Reaction system in the amount of 20 µL was used for qPCR with a StepOnePlus™ Real-Time PCR System (Applied Biosystems, Carlsbad, CA, USA), and the program was as follows: 94 °C for 30 s, followed by 40 cycles at 94 °C for 5 s, and 60 °C for 30 s. Relative transcript level of *NlPCE3* in different samples was evaluated using the 2^−∆∆Ct^ method.

### 2.3. dsRNA Synthesis and Injection

As with our previous methods [19], the DNA template of *NlPCE3* for dsRNA synthesis was 630 bp with primers ds*NlPCE3-*F and ds*NlPCE3-*R (Supplementary Table S1). dsRNA of the *GFP* gene (ds*GFP*) was taken as the negative control. The GFP gene sequence was synthesized in vitro based on the sequence of the binary vector pCAMBIA-1302 (GenBank: AF234298.1). The DNA template of *GFP* for dsRNA synthesis was 350 bp with primers ds*GFP*-F and ds*GFP*-R (Supplementary Table S1). ds*NlPCE3* and ds*GFP* were synthesized according to the instructions for a MEGAscript^TM^ T7 High Yield Transcription Kit (Thermo Fisher Scientific, Waltham, MA, USA). The concentration of synthesized dsRNA was determined using a NanoDrop 2000 spectrophotometer (Thermo Fisher Scientific). RNA interference was performed by injecting approximately 50 nL of dsRNA (5000 ng µL^−1^) into the abdomen of each 5th instar nymph using a manual microinjector. Injection of ds*NlPCE3* was made to 100 nymphs, and 100 nymphs received ds*GFP* as the negative control.

### 2.4. Observation of the Male Internal Genitalia and Fertility Analysis

After injection, once the 5th instar male nymphs emerged, one male was placed in a 50 mL centrifuge tube with two untreated virgin female adults and fed fresh rice seedlings. The seedlings were changed every day and the tube orifice was covered with gauze. The survival rate and the number of eggs laid by the untreated females were recorded and statistically analyzed. Subsequently, three-day-old male adults were dissected under a Nikon SMZ1500 stereozoom microscope (Nikon, Tokyo, Japan), and morphological changes were observed and photographed with the NIS Elements software (Nikon, Tokyo, Japan).

### 2.5. Immunofluorescence Staining

Immunofluorescence was used to analyze localization and distribution of target proteins in male internal genitalia under different treatments. All samples were washed three times in phosphate-buffered saline (PBS), then fixed with 4% paraformaldehyde in PBS for 2 h at room temperature. Immunofluorescence staining was performed as previously described [19,20]. The primary antibody was a rabbit polyclonal antibody against a *Nl*PCE3 polypeptide (VEGKSRHRRSIGDQ), and the secondary antibody was a goat anti-rabbit IgG antibody conjugated with Dylight 488 fluorescent dye (Abbkine, Wuhan, China). The samples were observed with laser scanning confocal microscopy (Leica SP8, Mannheim, Germany).

### 2.6. Data Analysis

Statistical analyses were conducted using one-way ANOVA followed by Tukey’s test for multiple comparisons and unpaired two-tailed Student’s *t*-tests using GraphPad Prism Software 8.0.2 (GraphPad Software, San Diego, CA, USA). All data are presented as the means ± standard error.

## 3. Results

### 3.1. Tissue-Specific Expression of NlPCE3

**Figure 1:**
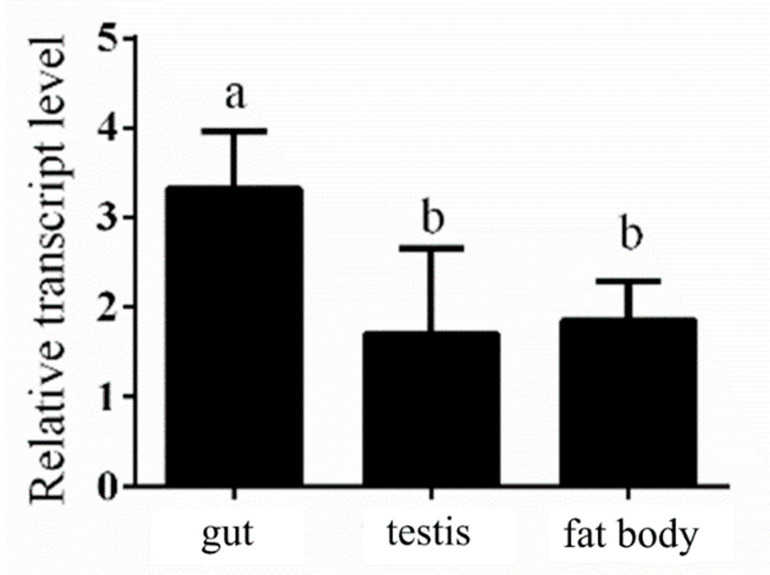
Expression of *NlPCE3* in different tissues of three-day-old male adults. The different tissues were dissected from 50 male individuals and divided into three pools for RNA extraction and qPCR analysis. Data are presented as the means ± standard error. Statistical analyses were conducted using one-way ANOVA and unpaired two-tailed Student’s *t*-test using GraphPad Prism Software 8.0.2 (GraphPad Software, San Diego, CA, USA). Lowercase letters indicate significant differences (*p* < 0.05).

Our previous experimental data showed that *NlPCE3* had a higher expression level in male adults than in female adults [19]. To further study the tissue-specific expression of *NlPCE3* in male adults, 50 three-day-old male adults were dissected and their guts, testes and fat bodies were collected for qPCR analysis. The results showed that *NlPCE3* had a higher expression level in the gut than in the fat body and testis (Figure 1).

### 3.2. Effect of RNAi on BPH Fecundity

**Figure 2:**
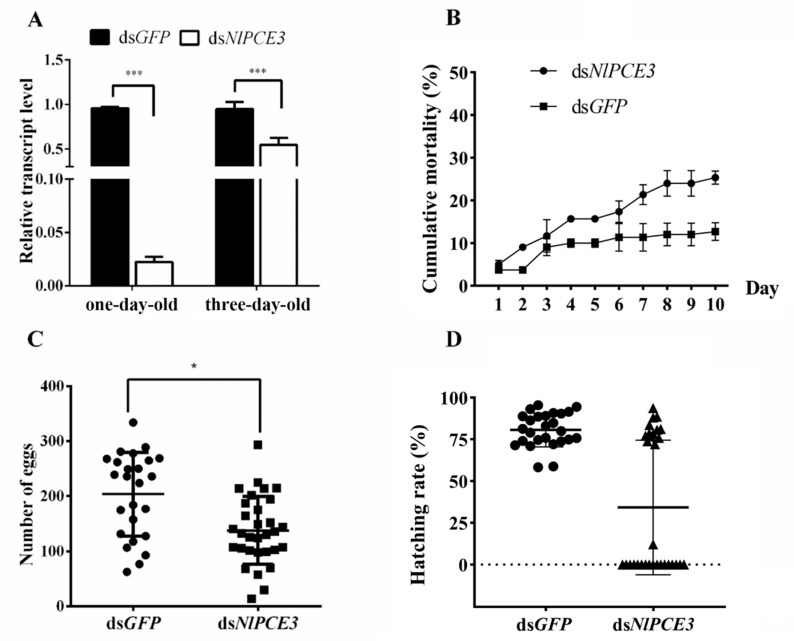
Effect of RNAi on BPH fecundity. (**A**) Downregulation of *NlPCE3* expression in one- and three-day-old male adults by dsRNA injection (three repetitions, five adults in one repetition). qPCR data of one- and three-day-old male subjects were analyzed independently. (**B**) Cumulative mortality of male BPH after ds*NlPCE3* and ds*GFP* injection (three repetitions, 20 individuals in one repetition). (**C**) The number of eggs laid by untreated females that mated with ds*NlPCE3- (n =* 31*)* and ds*GFP*-treated (*n* = 25) males. Each point on the graph represents the number of eggs laid by each female. (**D**) The hatching rate of eggs laid by untreated females that mated with ds*NlPCE3*-(*n* = 31) and ds*GFP*-treated (*n* = 23) males. Each point on the graph represents the hatching rate of eggs laid by each female. Data are presented as the means ± standard error. Statistical analyses were conducted using one-way ANOVA and unpaired two-tailed Student’s *t*-tests using GraphPad Prism Software 8.0.2 (GraphPad Software). In (A) and (C), * and *** represent significant differences of *p* < 0.05 and *p* < 0.001, respectively.

Following dsRNA injection, one- and three-day-old male adults were selected for qPCR detection of the RNAi effect on the *NlPCE3* expression level. We found that the transcriptional expression level of *NlPCE3* was prominently inhibited, especially in one-day-old males, indicating RNAi by dsRNA injection was effective (Figure 2A). The survival curve illustrated that the ds*NlPCE3* treatment group had a relatively higher mortality than the ds*GFP* treatment group, although this difference was not statistically significant (Figure 2B).

**Figure 3:**
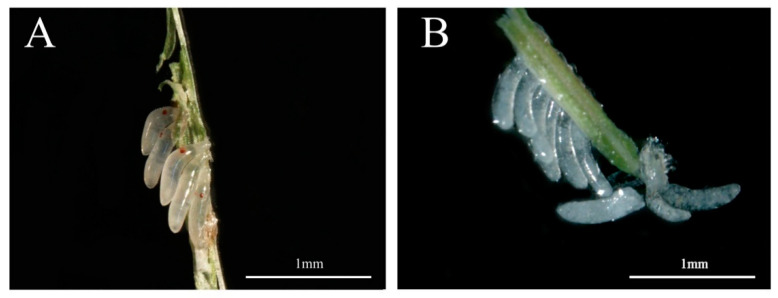
Five-day-old eggs. (**A**) Normal eggs laid by an untreated female that mated with a ds*GFP-*treated male. (**B**) Abnormal eggs laid by an untreated female that mated with a ds*NlPCE3-*treated male. The abnormal eggs laid by untreated females that mated with ds*NlPCE3-*treated males are unable to hatch and lack eye-spots.

Further analysis revealed that the mean number of eggs laid by untreated female adults that mated with ds*NlPCE3*-treated male adults was less than that of the eggs laid by the females that mated with ds*GFP*-treated male adults (Figure 2C). In addition, the hatching rate of eggs in the ds*NlPCE3* treatment group was reduced. Among the 31 females that mated with ds*NlPCE3*-treated males, 17 females laid eggs that could not hatch (Figure 2D), and no eye-spot (as described by Fan et al. (2020) [21]) was found on these non-viable eggs (Figure 3). 

### 3.3. Effect of RNAi on Male Internal Genitalia and Sperm

**Figure 4:**
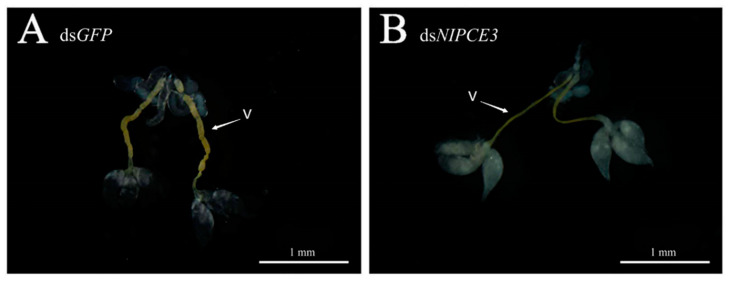
Effect of RNA interference on the internal genitalia development of a three-day-old male adult. (**A**) The internal genitalia of a ds*GFP*-treated male. (**B**) The internal genitalia of a ds*NlPCE3*-treated male. V: vas deferens.

**Figure 5:**
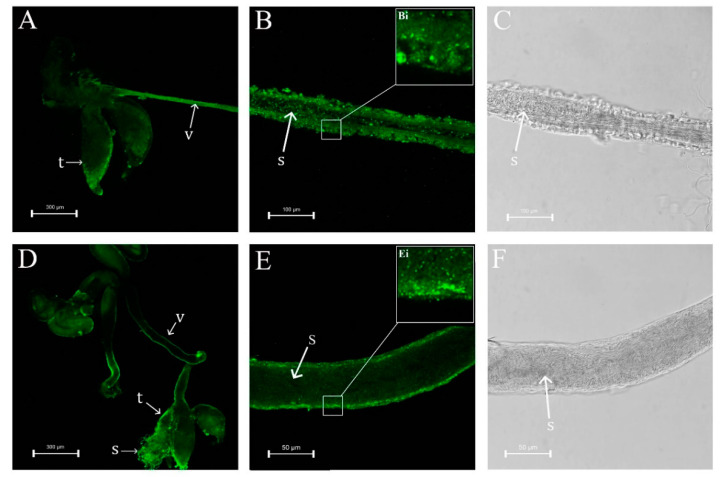
Effect of RNAi on *NlPCE3* expression in the internal genitalia of a three-day-old male adult. (**A**) The internal genitalia of a male treated with ds*NlPCE3*. (**B**,**C**) The vas deferens of a male treated with ds*NlPCE3.* (**D**) The internal genitalia of a male treated with ds*GFP*. (**E**,**F**) The vas deferens of a male treated with ds*GFP.* t: testis, v: vas deferens, s: sperm.

Observation of male internal genitalia revealed that the vas deferens of ds*NlPCE3*-treated males was markedly thinner than that of ds*GFP-*treated males (Figure 4 and Figure 5). Immunofluorescence staining showed that *Nl*PCE3 was widely expressed in male internal genitalia (Figure 5A,D), but after ds*NlPCE3* treatment, expression of *Nl*PCE3 was diffuse in the vas deferens of ds*NlPCE3*-treated males (Figure 5B,E). In addition, the peripheral cells of ds*NlPCE3*-treated males’ internal genitalia seemed degraded (Figure 5Bi,Ei). However, the size of the testes appeared not affected by ds*NlPCE3* treatment.

**Figure 6:**
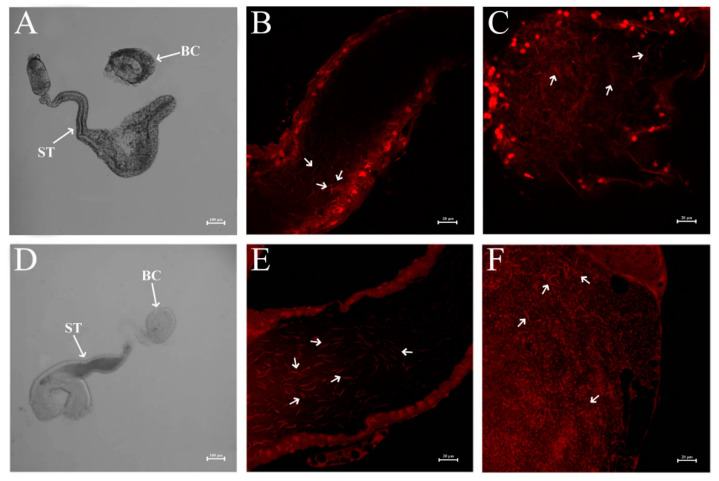
Sperm in the spermatheca and bursa copulatrix. (**A**) Spermatheca and bursa copulatrix of an untreated female that mated with ds*GFP*-treated males. (**B**) Sperm in the spermatheca of an untreated female that mated with ds*GFP*-treated males. (**C**) Sperm in the bursa copulatrix of an untreated female that mated with ds*GFP*-treated males. (**D**) Spermatheca and bursa copulatrix of an untreated female that mated with ds*NlPCE3-*treated males. (**E**) Sperm in the spermatheca of an untreated female that mated with ds*NlPCE3-*treated males. (**F**) Sperm in the bursa copulatrix of an untreated female that mated with ds*NlPCE3-*treated males. Nuclei were stained with propidium iodide. ST: spermatheca, BC: bursa copulatrix. White arrows indicate sperm in (**B**,**C**,**E**,**F**).

Furthermore, we investigated the effect of ds*NlPCE3* on sperm. We found sperm in the vas deferens was active, and there was a large number of sperm in the testes of ds*NlPCE3-*treated males (Figure 5C,F) and in the spermathecae and bursae copulatrix of untreated female adults that mated with ds*NlPCE3-*treated males (Figure 6).

## 4. Discussion

In internally fertilizing species, seminal fluid proteins (SFPs) are transferred with sperm from males to their mates during copulation. These proteins play significant roles in the process of reproduction, including sperm capacitation, sperm storage and competition and fertilization; they can also influence female behavior and physiology in some cases [22,23,24]. Proteolysis regulators (proteases and their inhibitors) are a component of the seminal fluid of many animals regulating functions of the proteins necessary for male or female reproduction by degradation or removal of inhibitory peptides or through processing of prohormones [25]. The predicted proteolysis regulators in humans and insects mainly include SPs, cysteine proteases, threonine proteases and SP inhibitors [26]. The SPs and SP inhibitors are the most common types of proteases and protease inhibitors, respectively [25].

In insects, SFPs have been proven to be one of the seminal fluid substances involved in the regulation of reproduction. Females have higher levels of oogenesis and oviposition when they receive SFPs [27]. SFPs of the SP family have been detected in male gonads of *D. melanogaster*, and these proteins affect female oviposition through proteolysis cascade regulation [28]. SPs in males were demonstrated to induce oviposition by females, with SPs and SP inhibitors playing a key role in the phenotypic changes between pre-fertilization and post-mating eggs [29]. *D. melanogaster* females that mated with males whose seminase (a predicted SP) was knocked down by RNAi failed to maintain a high level of oviposition [25]. In *Allonemobius socius*, RNAi knockdown of the ejaculate serine protease in males weakened their ability to induce female oviposition [30]. SPs are also an energy source of sperm motility, inducing sperm movement [31]. In *M. sexta*, sperm motility depends on the proteases secreted from the male reproductive tract [32]. In *Aquarius remigis*, a trypsase plays important roles in activating the signal pathway of sperm motility [33].

In the present study, we found that *NlPCE3* plays an important role in the reproduction of male BPH. First, we found that female adults that mated with ds*NlPCE3-*treated male adults laid fewer eggs than those that mated with ds*GFP*-treated males, and more than half of these eggs could not hatch and lacked eye-spots. However, we also found a large amount of sperm in the spermathecae and bursae copulatrix of the mating females. These results demonstrate that although sperm could still be transmitted from a male to a female after ds*NlPCE3* treatment, male reproductive capability was affected. *Nl*PCE3 belongs to the clip-SP family, which has only been identified in arthropods [1]. The catalytically active structure of *Nl*PCE3 is the C-terminal SP domain, and the clip domain’s function may be to regulate the activity of SP in the last step of the insect SP pathway [3,7]. *Nl*PCE3 may participate in sperm capacitation, increasing the odds of successful fertilization. Alternatively, *NlPCE3* may participate in the functional regulation of SFPs of male BPH as a proteolysis regulator, enhancing the ability of male-to-female oviposition induction. Second, we also observed that the male vas deferens became thinner after RNAi, and the peripheral cells seemed degraded. Even though the size of the testes appeared not affected by ds*NlPCE3* treatment, it cannot be excluded that the ds*NIPCE3*-treated males had delayed sexual maturation or development, since the vas deferens was thinner. It is questioned whether this weakness of the vas deferens might affect transmission or ejaculation efficiency of the sperm. We tried to compare the amount of sperm produced by ds*GFP*-treated and ds*NIPCE3*-treated males, but, regrettably, there is no effective method to quantify the amount of sperm. In female BPH, we also found that the follicular cells outside the terminal follicle were destroyed when the expression of *NlPCE3* in follicular cells was disrupted by RNAi, and ovarian development was prominently inhibited [19]. Taken together, these results indicate that *NlPCE3* affects development of both male and female internal genitalia. Since *NlPCE3* is so important for BPH development, it is a possible target for controlling BPH.

## 5. Conclusions

In general, although the main functions of clip-SPs in insects have been studied, a full understanding of the role of clip-SPs remains incomplete. In a previous study, we found *NlPCE3* was involved in egg production of female BPH [19]. In the present study, we show that *NlPCE3* also plays an important role in the reproduction of male BPH. These results provide important evidence for revealing more functions of clip-SPs in insects. However, the tissue-specific expression of *NlPCE3* differs between adult female and male BPH. In adult females, *NlPCE3* expression is higher in the gut and fat body than in the ovaries, but in adult males, it is higher in the gut than in the fat body and testis. The reason for this difference between the sexes requires further study. Furthermore, as *Nl*PCE3’s specific molecular interaction mechanism in BPH remains unknown, future work will focus on identifying the interacting proteins of *Nl*PCE3 to reveal their interaction mechanism during the PCE3 functioning in BPH reproduction.

## Supplementary Materials

The following are available online at https://www.mdpi.com/2075-4450/12/2/114/s1, Table S1: The primers used in this study.
