# Supplementary material for: PCE3 Plays a Role in the Reproduction of Male Nilaparvata lugens
Source: Insects. 2021 Jan 28;12(2):114. doi: 10.3390/insects12020114 (PMC7911326; doi:10.3390/insects12020114)
Supplement: Supplementary file 1 [file insects-12-00114-s001.pdf]

Table S1. The primers used in this study [1]

Primers	Primer Sequence (5′–3′)
q*NlPCE3*-F	CGAAATGGAAGATTGCTGAGTC
q*NlPCE3*-R	TGGTGTTGGCGTTGATTATGG
q*Nl*18s-F	GTAACCCGCTGAACCTCC
q*Nl*18s-R	GTCCGAAGACCTCACTAAATCA
ds*NlPCE3-F*	GGATCCTAATACGACTCACTATAGGGACTCTCGTTTCCAGATACC
ds*NlPCE3-R*	GGATCCTAATACGACTCACTATAGGCACCAGTGACTTCGCTC
ds*GFP*-F	GGATCCTAATACGACTCACTATAGGGATACGTGCAGGAGAGGAC
ds*GFP*-R	GGATCCTAATACGACTCACTATAGGGCAGATTGTGTGGACAGG

Reference

1. Wu, J.M.; Zheng, R.E.; Zhang, R.J.; Ji, J.L.; Yu, X.P.; Xu, Y.P. A clip domain serine protease involved in egg production in *Nilaparvata lugens*: Expression patterns and RNA interference. *Insects* **2019**, *10*, 378.
